# Peer tobacco use, media exposure, and support for tobacco control policies among medical and public health students in Georgia: A cross-sectional study

**DOI:** 10.18332/tid/226286

**Published:** 2026-08-06

**Authors:** Tinatin Mamatsashvili, Levan Gabelaia, Levan Baramidze, Nino Kiladze, Nino Gvajaia

**Affiliations:** 1 Department of Public Health, Tbilisi State Medical University, Tbilisi, Georgia; 2 Department of Public Health, University of Georgia, Tbilisi, Georgia

**Keywords:** tobacco use, tobacco control, medical students, peer influence, Georgia

## Abstract

**INTRODUCTION:**

Health professional students are potential tobacco users and future advocates for tobacco control. Evidence from the Caucasus on factors associated with tobacco use and policy support remains limited. This study examined tobacco use and support for tobacco-control policies among medical and public health students in Georgia.

**METHODS:**

A cross-sectional survey was conducted from June to December 2022 among students at Tbilisi State Medical University using a self-administered online questionnaire; all data were self-reported. Of 1078 randomly invited students, 667 completed the survey. Any past 30-day use of cigarettes, electronic cigarettes, heated tobacco products, cigars, bidis, or hookah was assessed, together with tobacco-related media exposure, peer and household tobacco use, and support for five tobacco-control policies. Multivariable logistic and linear regression examined factors associated with current use and policy support, adjusting for sociodemographic characteristics, media and retail exposures, and social exposures.

**RESULTS:**

Overall, 18.6% of students reported past 30-day tobacco or nicotine product use, and mean policy support was 5.45 on a 7-point scale. In adjusted analyses, the number of friends who used tobacco was the factor most strongly associated with current use (adjusted odds ratio, AOR=2.49; 95% CI: 1.81–3.43). Anti-tobacco media exposure was modestly associated with higher odds of use (AOR=1.07; 95% CI: 1.02–1.11), whereas pro-tobacco exposure was inversely associated (AOR=0.92; 95% CI: 0.87–0.97). Current use (adjusted regression coefficient, B= -0.65; 95% CI: -0.94 – -0.35) and male sex (B= -0.41; 95% CI: -0.64 – -0.18) were independently associated with lower policy support.

**CONCLUSIONS:**

Peer tobacco use was the factor most strongly associated with current tobacco or nicotine product use among medical and public health students in Georgia. Although policy support was generally high, current users expressed weaker support. Additional longitudinal and multi-institutional studies are needed to provide sufficient evidence to inform tobacco-use prevention and the promotion of tobacco-control engagement during health professional training.

## INTRODUCTION

Tobacco use remains a major preventable cause of morbidity and mortality worldwide and continues to contribute substantially to the global burden of non-communicable diseases^1,2^. Prevention efforts are especially important before tobacco or nicotine use becomes established, as most daily smokers initiate use before early adulthood^3^. University students therefore represent a key population for tobacco-control research, particularly because the transition into young adulthood is marked by greater independence, changing social networks, and increased exposure to peer norms that may influence tobacco and nicotine product use^4,5^.

Health professional students are a particularly important subgroup within this population. As future physicians, public health specialists, and health advocates, their personal tobacco-related behaviors may influence not only their own health but also their future confidence in delivering cessation counseling, supporting smoke-free environments, and promoting evidence-based tobacco-control policies^6,7^. At the same time, these students remain susceptible to the same social and environmental influences as other young adults, including peer tobacco use, tobacco-related media exposure, and visibility of tobacco products in retail or social settings^4,5^. Understanding tobacco use in this group is therefore relevant both for student health and for the development of future tobacco-control capacity.

Peer influence is one of the most consistently described determinants of tobacco and nicotine use among adolescents and young adults^5,8^. In university settings, peer norms may be especially influential because students often spend substantial time in shared academic and social environments. Tobacco use among friends may normalize use, reduce perceived harm, and increase opportunities for initiation or continuation^8,9^. In contrast, family or household exposure may become less influential during young adulthood as peer identity and social belonging become more central^10^. For medical and public health students, this distinction may be important for understanding the social context in which tobacco or nicotine product use occurs during health professional training.

Media exposure may also be associated with tobacco-related behavior and attitudes, although its role is complex. Pro-tobacco media and promotional content may increase product visibility and perceived acceptability, particularly for newer nicotine products such as electronic cigarettes and heated tobacco products^11^. Anti-tobacco media campaigns, warning messages, and cessation-oriented content are intended to reduce use and strengthen tobacco-free norms^12,13^. However, associations between media exposure and tobacco use in cross-sectional studies may be difficult to interpret because current users may be more likely to notice or recall tobacco-related messages. Therefore, examining both pro- and anti-tobacco exposure alongside peer influences may provide a more complete understanding of the tobacco-related environment experienced by health professional students.

In addition to tobacco use itself, support for tobacco-control policies is an important outcome. Public support can influence the feasibility, implementation, and sustainability of tobacco-control measures^12,14^. Previous evidence suggests that people who use tobacco are often less supportive of restrictive tobacco-control policies than non-users^15^. Among future healthcare and public health professionals, this relationship may have broader implications. Students who personally use tobacco or who are embedded in tobacco-using peer networks may be less supportive of policies that restrict tobacco availability, advertising, or use in public spaces. Conversely, strong policy support among health professional students may reflect developing professional identity and alignment with public health goals^7^.

These questions are particularly relevant in Georgia, a country in the Caucasus region with a historically high burden of tobacco use and evolving tobacco-control legislation^16^. Although tobacco-control policies have strengthened^16^, less is known about how young health professionals in training perceive tobacco use and tobacco regulation. Evidence from this population is limited, despite their future role in clinical counseling, public health practice, and policy advocacy. Studying medical and public health students in Georgia may therefore provide useful insight into whether tobacco-control norms are becoming embedded among future health professionals and where university-level prevention efforts should be strengthened.

This study examined tobacco and nicotine product use, tobacco-related media exposure, peer and household tobacco exposure, and support for tobacco-control policies among medical and public health students in Georgia. We hypothesized that stronger peer tobacco-use norms would be associated with higher odds of current tobacco or nicotine product use, and that current tobacco use would be associated with lower support for tobacco-control policies. We also explored the associations of pro- and anti-tobacco media exposure with both current use and policy support.

## METHODS

### Study design, setting, and participants

This cross-sectional study used survey data collected from June to December 2022 among students at Tbilisi State Medical University, a public university and the largest medical university in Georgia. Eligible participants were students enrolled in one of four academic programs: Georgian-language MD, English-language MD, Bachelor of Public Health, or Master of Public Health. At the time of data collection, 6342 students were enrolled in these eligible programs.

A random sample of 1078 eligible students was selected from the official university enrollment registry using a computerized random number generator, ensuring equal probability of selection across eligible academic programs. Selected students were invited to participate by email or during face-to-face recruitment. A total of 667 students completed the survey, corresponding to a response rate of 61.8%. Because data were collected anonymously, differences between respondents and non-respondents could not be assessed; therefore, non-response bias cannot be excluded.

### Ethics

Ethical approval was obtained from the Tbilisi State Medical University Biomedical Research Ethics Committee (December 2021, No. 9-2021/93). Participants were informed about the study before starting the questionnaire, and informed consent was obtained prior to data collection. Data were collected anonymously.

### Survey instrument

The questionnaire was developed in Georgian and English and administered online. Before full deployment, it was pilot-tested among 20 students to assess clarity, readability, and comprehensibility. Minor wording changes were made after the pilot phase. The final questionnaire assessed sociodemographic characteristics, tobacco and nicotine product use, tobacco-related media exposure, social exposure to tobacco use, and support for tobacco-control policies. All data were self-reported.

### Measures

Sociodemographic variables included age, sex (female or male), nationality (native Georgian or other nationality), and academic program (Georgian-language MD, English-language MD, Bachelor of Public Health, or Master of Public Health). Sociodemographic characteristics, media and retail exposures, and social exposure variables were considered potential confounding factors of the associations of interest and were therefore included as covariates in the multivariable models described below.

The primary behavioral outcome was any past 30-day tobacco or nicotine product use. Participants were asked how many days during the past 30 days they had used cigarettes, electronic cigarettes, heated tobacco products (IQOS), cigars, bidis, or hookah. A dichotomous variable was created to indicate any past 30-day use of at least one tobacco or nicotine product (1= use of at least one product; 0=no use).

Tobacco-related media exposure was assessed by asking participants how many days during the past 30 days they had noticed anti-tobacco messages, such as information about health risks or encouragement to quit, and pro-tobacco content, such as advertisements, promotional signs, or tobacco-related displays, including exposure at points of sale. Composite anti-tobacco and pro-tobacco media exposure indices were created by averaging responses across relevant items, with higher scores indicating greater exposure, and were analyzed as continuous variables.

Social exposure to tobacco use was assessed by asking participants how many of their friends used tobacco or nicotine products (analyzed as a continuous variable) and whether any household member other than the participant used tobacco products (yes, no).

Support for tobacco-control policies was measured using five statements addressing cigarette taxation, smoke-free indoor laws, smoking in cars with children, elimination of flavored tobacco products, and increasing the legal age for tobacco purchase to 21 years. Responses were recorded on a 7-point Likert scale ranging from 1 (strongly disagree) to 7 (strongly agree). An overall tobacco-control policy support score was calculated by averaging responses across the five items, with higher values indicating stronger support.

### Statistical analysis

Descriptive statistics were used to summarize participant characteristics, tobacco and nicotine product use, tobacco-related media and retail exposure, social exposure variables, and tobacco-control policy support. Bivariate analyses were conducted to compare characteristics by any past 30-day tobacco or nicotine product use status and to examine associations with policy support. Specifically, chi-squared tests were used for categorical variables, independent-samples t-tests were used to compare continuous variables by past 30-day use status, and Pearson correlation coefficients (r) were used to examine associations between continuous variables and the tobacco-control policy support index score.

Multivariable binary logistic regression was used to examine factors associated with any past 30-day tobacco or nicotine product use. Covariates included age, sex, nationality, anti-tobacco media exposure, pro-tobacco media exposure, retail exposure, number of friends who used tobacco, and presence of other smokers in the household. Because all odds ratios were adjusted for the covariates listed above, they are reported as adjusted odds ratios (AORs) with 95% confidence intervals (CIs) and p-values.

Multivariable linear regression was used to examine factors associated with tobacco-control policy support. The model included sociodemographic variables, media and retail exposure variables, social exposure variables, and any past 30-day tobacco or nicotine product use. Unstandardized regression coefficients (B), adjusted for all other variables in the model, 95% CIs, and p-values are reported. Cases with missing data on variables included in regression models were excluded using listwise deletion. Before model estimation, linear regression assumptions, including normality of residuals, homoscedasticity, and absence of multicollinearity, were assessed. Statistical analyses were conducted using SPSS version 27 (IBM Corp., Armonk, NY, USA), with statistical significance set at α=0.05.

## RESULTS

A total of 667 students completed the survey. The sample included 449 female students (68.9%) and 203 male students (31.1%), with a mean age of 21.41 years (SD=2.67). Overall, 307 participants (46.0%) identified as Georgian and 360 (54.0%) as other nationalities. Participants were enrolled in the Georgian-language MD program (n=180; 27.0%), English-language MD program (n=295; 44.2%), Bachelor of Public Health program (n=153; 22.9%), and Master of Public Health program (n=39; 5.8%).

Overall, 37.8% of participants reported ever trying cigarettes, 24.1% electronic cigarettes, 13.0% heated tobacco products, 12.7% hookah, 9.4% cigars, and 6.3% bidis; 43.9% reported never trying any tobacco product. The prevalence of any past 30-day tobacco or nicotine product use was 18.6%. Product-specific past 30-day use was 15.3% for cigarettes, 7.6% for electronic cigarettes, 3.9% for heated tobacco products, 3.0% for hookah, 1.8% for cigars, and 1.6% for bidis. The mean pro-tobacco exposure index score was 8.73 (SD=13.75), and the mean anti-tobacco exposure index score was 5.55 (SD=7.66).

In bivariate analyses (Table 1), past 30-day tobacco or nicotine product use was associated with older age, male sex, Georgian nationality, greater anti-tobacco media exposure, lower retail exposure, more friends who used cigarettes, and having other smokers in the household. Tobacco-control policy support was lower among students who reported past 30-day tobacco or nicotine product use than among non-users (mean: 4.90 vs 5.58; mean difference= -0.68; 95% CI: -0.90 – -0.46; p<0.001).

**Table 1:** Participant characteristics, bivariate associations with past 30-day tobacco or nicotine product use, and tobacco-control policy support among medical and public health students in a cross-sectional study, Tbilisi State Medical University, Georgia, 2022 (N=667)

*Characteristics*	*Total* *n (%)*	*No past 30-day* *tobacco use* *n (%)*	*Any past 30-day* *tobacco use* *n (%)*	*p*	*Policy* *support index* *score* *Mean (SD)*	*p*
**Total**	667 (100)	536 (81.4)	131 (18.6)			
**Demographic factors**						
**Age** (years), mean (SD)	21.41 (2.67)	21.27 (2.74)	21.98 (2.33)	0.007	r=0.02	0.673
**Sex**						<0.001
Female	449 (68.9)	376 (72.0)	73 (56.2)	0.001	5.57 (0.73)	
Male	203 (31.1)	146 (28.0)	57 (43.8)		5.15 (1.30)	
**Native Georgian**				0.031		
No	307 (46.0)	258 (48.1)	49 (37.4)		5.66 (1.22)	0.001
Yes	360 (54.0)	278 (51.9)	82 (62.6)		5.30 (0.96)	
**Academic program**				0.282		
Georgian MD	180 (27.0)	140 (26.1)	40 (30.5)		5.16 (0.95)	0.001
English MD	295 (44.2)	247 (46.1)	48 (36.6)		5.65 (1.22)	
Bachelor of Public Health	153 (22.9)	119 (22.2)	34 (26.0)		5.39 (0.91)	
Master of Public Health	39 (5.8)	30 (5.6)	9 (6.9)		5.65 (1.03)	
***Media and retail exposure (days in past 30 days)***	***Mean (SD)***	***Mean (SD)***	***Mean (SD)***		***r***	
Anti-tobacco: TV, radio, social media, billboards	6.14 (8.91)	5.80 (8.79)	7.59 (9.32)	0.107	0.02	0.673
Anti-tobacco: elsewhere	4.90 (7.78)	4.40 (7.28)	7.00 (9.37)	0.008	−0.05	0.758
Anti-tobacco media exposure index score	5.55 (7.66)	5.13 (7.48)	7.31 (8.21)	0.023	−0.01	0.386
Pro-tobacco: TV, social media, billboards	4.58 (7.80)	4.72 (7.99)	3.96 (7.00)	0.442	0.01	0.800
Pro-tobacco: elsewhere	4.11 (6.90)	4.34 (7.26)	3.15 (5.06)	0.174	0.01	0.996
Pro-tobacco media exposure index score	8.73 (13.75)	9.12 (14.29)	7.11 (11.21)	0.248	0.01	0.975
Notice tobacco ads/displays	4.05 (1.98)	4.19 (1.93)	3.44 (2.08)	0.002	0.32	<0.001
Notice tobacco special stores	4.41 (1.90)	4.47 (1.92)	4.16 (1.78)	0.199	0.32	<0.001
Retail exposure index score	4.22 (1.66)	4.32 (1.65)	3.80 (1.65)	0.011	0.38	<0.001
**Social exposures**						
Number of friends who use cigarettes	2.55 (1.06)	2.36 (0.98)	3.39 (1.01)	0.001	−0.07	0.158
	***n (%)***	***n (%)***	***n (%)***		***Mean (SD)***	
**Other smokers in the home**				0.001		0.128
No	301 (57.3)	256 (61.0)	45 (42.9)		5.52 (1.09)	
Yes	224 (42.7)	164 (39.0)	60 (57.1)		5.36 (1.10)	
	***Mean (SD)***	***Mean (SD)***	***Mean (SD)***			
**Tobacco-control policy support** (1 – 7 scale)						
Cigarette taxation	4.42 (2.05)	4.61 (1.97)	3.58 (2.20)	<0.001		
Prohibiting flavored products	4.13 (1.97)	4.16 (1.93)	4.01 (2.15)	0.541		
Tobacco 21 (T21)	5.74 (1.84)	5.86 (1.74)	5.22 (2.15)	0.005		
Smoke-free indoor policy	6.27 (1.54)	6.50 (1.28)	5.29 (2.07)	<0.001		
Smoke-free cars with children	6.69 (1.07)	6.74 (1.00)	6.48 (1.30)	0.045		
Policy support index score	5.45 (1.09)	5.58 (1.02)	4.90 (1.21)	<0.001		

Pearson correlation coefficient (r) between the characteristic and the policy support index score. Policy support was measured on a 7-point scale (1=strongly disagree to 7=strongly agree). Percentages may not sum to 100% because of missing data. P-values compare groups by past 30-day tobacco or nicotine product use status (column 5) and by tobacco-control policy support (column 7); p-values are from chi-squared tests (categorical variables), independent-samples t-tests (continuous variables), or tests of the Pearson correlation coefficient.

In multivariable logistic regression (Table 2), adjusted for age, sex, nationality, anti- and pro-tobacco media exposure, retail exposure, number of friends who used cigarettes, and other smokers in the household, number of friends who used cigarettes was the factor most strongly and independently associated with any past 30-day tobacco or nicotine product use (AOR=2.49; 95% CI: 1.81–3.43; p<0.001). Anti-tobacco media exposure was modestly associated with higher odds of current use (AOR=1.07; 95% CI: 1.02–1.11; p=0.004), whereas pro-tobacco media exposure was associated with lower odds of current use (AOR=0.92; 95% CI: 0.87–0.97; p=0.002). Retail exposure was borderline but not statistically significant in the adjusted model (AOR=0.83; 95% CI: 0.69–1.01; p=0.052).

**Table 2:** Multivariable logistic and linear regression models of factors associated with past 30-day tobacco or nicotine product use and tobacco-control policy support among medical and public health students in a cross-sectional study, Tbilisi State Medical University, Georgia, 2022 (N=667)

*Variables*	*Any past 30-day tobacco or nicotine product* *use*			*Tobacco-control policy support index score*		
	*AOR*	*95% CI*	*p*	*B*	*95% CI*	*p*
**Demographic factors**						
Age (years)	1.05	0.93–1.19	0.427	0.05	0.004–0.10	0.033
Male (ref: female)	1.80	0.97–3.32	0.061	−0.41	−0.64 – -0.18	<0.001
Native Georgian (ref: other nationality)	1.31	0.69–2.48	0.409	−0.22	−0.45–0.01	0.056
**Media and retail exposures**						
Anti-tobacco media exposure (continuous)	1.07	1.02–1.11	0.004	0.01	−0.01–0.02	0.523
Pro-tobacco media exposure (continuous)	0.92	0.87–0.97	0.002	−0.01	−0.03–0.01	0.187
Retail exposure index (continuous)	0.83	0.69–1.01	0.052	0.21	0.15–0.28	<0.001
**Social exposures**						
Number of friends who use cigarettes (continuous)	2.49	1.81–3.43	<0.001	0.03	−0.08–0.15	0.554
Other smokers in the home (ref: no)	0.93	0.50–1.72	0.808	0.06	−0.16–0.28	0.597
Any past 30-day tobacco use (ref: no)			−0.65	−0.94 – -0.35	<0.001	
**Model fit**	Nagelkerke R^2^=0.279			Adjusted R^2^=0.212		

AOR: adjusted odds ratio; adjusted odds ratios are from multivariable binary logistic regression. B: unstandardized regression coefficient from multivariable linear regression. Each estimate is adjusted for all other variables listed in the table. Age, media exposure indices, the retail exposure index, and number of friends who use cigarettes were modeled as continuous variables and therefore have no reference category; estimates represent the change in odds (AOR) or in the policy support score (B) per one-unit increase. Nagelkerke R^2^=0.279 for the logistic model; adjusted R^2^=0.212 for the linear model.

In multivariable linear regression (Table 2), with all coefficients adjusted for the other variables in the model, current tobacco or nicotine product use was independently associated with lower tobacco-control policy support (B= -0.65; 95% CI: -0.94 – -0.35; p<0.001). Male sex was associated with lower policy support compared with female sex (B= -0.41; 95% CI: -0.64 – -0.18; p<0.001). Older age (B=0.05; 95% CI: 0.004–0.10; p=0.033) and greater retail exposure (B=0.21; 95% CI: 0.15–0.28; p<0.001) were associated with stronger policy support.

## DISCUSSION

In this study of medical and public health students in Georgia, three findings are most important. First, nearly one in five students reported past 30-day tobacco or nicotine product use. Second, peer tobacco use was the factor most strongly associated with current use. Third, although tobacco-control policy support was generally high, current tobacco or nicotine product use was associated with weaker policy support.

The prevalence of past 30-day tobacco or nicotine product use in this sample was lower than national adult tobacco estimates in Georgia^16^, but it remains important because these students are future healthcare and public health professionals. Tobacco use among health professionals has implications beyond individual behavior, as clinicians who smoke may be less likely to provide cessation counseling or may feel less confident discussing tobacco use with patients^6^. Therefore, tobacco use in this group should be considered not only a student health issue but also a future workforce and tobacco-control capacity issue.

Peer tobacco use showed the largest association with current tobacco or nicotine product use. Each increase in the number of friends who used cigarettes was associated with more than twice the odds of current use. This finding is consistent with social network theory, which suggests that smoking behaviors cluster within social networks rather than occurring only as isolated individual choices^8^. Longitudinal studies of adolescents and young adults also support the importance of social selection and social influence, whereby individuals may both choose peers with similar behaviors and be influenced by peer behavior over time^5,9^. Additional longitudinal studies in health professional student populations are needed to clarify how peer networks relate to tobacco and nicotine product use during training.

In contrast, household tobacco exposure was not independently associated with current use in the adjusted model. During young adulthood, peer identity and social belonging may become more influential than family modeling, particularly in university environments where students spend substantial time in shared academic and social settings^10^. This does not mean that household exposure is unimportant, but it suggests that the immediate peer environment may be particularly relevant to tobacco or nicotine product use in this population; further studies are needed to confirm this pattern.

The findings regarding media exposure require cautious interpretation. Anti-tobacco media exposure was positively associated with current use, but the magnitude of this association was small compared with peer influence. In a cross-sectional study, this finding should not be interpreted as evidence that anti-tobacco messages increase tobacco use. A more plausible explanation is selective attention or recall: students who use tobacco may be more likely to notice or remember tobacco-related warnings and cessation messages^17^. Psychological reactance may also occur when some individuals perceive directive health messages as threatening autonomy, although this cannot be tested with the present data^18^.

The inverse association between pro-tobacco media exposure and current use was also unexpected and should not be interpreted as a protective effect. This pattern may reflect measurement limitations, residual confounding, differences in where students spend time, or more critical processing of promotional content among health professional students. Prior evidence suggests that the effects of tobacco-related media may depend on educational context, health literacy, prior beliefs, and product-specific susceptibility^13^. Future studies should use longitudinal designs and more detailed measures of product-specific advertising exposure, including social media, point-of-sale marketing, and newer nicotine product promotion.

Policy support was generally high, particularly for smoke-free environments and restrictions on smoking in cars with children. However, students who reported current tobacco or nicotine product use expressed substantially lower support for tobacco-control policies. This finding is consistent with evidence from European populations showing that smokers tend to be less supportive of restrictive smoke-free policies than non-smokers^19^. Such differences are often interpreted through cognitive dissonance or policy self-interest frameworks, whereby individuals may resist regulations that constrain their own behavior^20^.

Sex differences in policy support were also observed. Male students reported lower policy support than female students in the adjusted model, and bivariate results showed stronger support among female students. Similar sex differences in support for smoke-free policies have been reported in other settings, where women often show higher perceived risk and stronger support for protective legislation^21^. Older age was also modestly associated with stronger policy support, which may reflect professional maturation and greater alignment with public health norms during training^7^.

These findings may be relevant for medical universities in countries where tobacco-control policies are still being consolidated. Because these students will later be involved in patient counseling, institutional norms, and public health practice, the patterns observed here warrant further investigation. However, given the cross-sectional design of this study, additional longitudinal and interventional studies are needed to provide sufficient evidence on whether, and how, tobacco use and policy support among health professional students can be modified and whether such changes influence later professional practice.

### Strengths and limitations

The study has several strengths. It included a relatively large sample of medical and public health students, used random selection from eligible academic programs, assessed multiple tobacco and nicotine products, and examined both behavioral and policy-attitudinal outcomes. It also provides evidence from Georgia and the Caucasus region, where student-level tobacco-control research remains limited.

Several limitations should also be considered. First, the cross-sectional design precludes causal inference, particularly for the associations involving media exposure. Second, tobacco and nicotine product use was self-reported, which may introduce misclassification of the outcome due to recall or social desirability bias. Third, the sample was drawn from one institution, which limits generalizability to all Georgian university students or all health professional students. Fourth, because the survey was anonymous, differences between respondents and non-respondents could not be assessed. Fifth, the study did not measure potentially relevant confounding factors such as stress, nicotine dependence, perceived harm, professional identity formation, or detailed product-specific susceptibility; therefore, residual confounding cannot be excluded. Future longitudinal and multi-institutional studies would help clarify causal pathways and provide sufficient evidence to guide future work in this population.

## CONCLUSIONS

Among medical and public health students in Georgia, past 30-day tobacco or nicotine product use was reported by a meaningful minority of participants. Peer tobacco use was the factor most strongly associated with current use, while current use was associated with lower support for tobacco-control policies. Additional longitudinal and multi-institutional studies are needed to provide sufficient evidence on the pathways underlying these associations and on how tobacco-control engagement develops during health professional training.

## Data Availability

The data supporting this research are available from the authors on reasonable request.
